# Isolation and characterization of *Corynebacterium* spp. from bulk tank raw cow's milk of different dairy farms in Germany

**DOI:** 10.1371/journal.pone.0194365

**Published:** 2018-04-04

**Authors:** Julia Hahne, Tabea Kloster, Sandra Rathmann, Mareike Weber, André Lipski

**Affiliations:** Rheinische Friedrich-Wilhelms-University Bonn, Institution of Nutrition and Food Science, Department of Food Microbiology and Hygiene, Bonn, North Rhine-Westphalia, Germany; Universidad Nacional Autonoma de Mexico Facultad de Quimica, MEXICO

## Abstract

We detected *Corynebacterium* spp. in raw milk samples of three farms by means of a selective, tellurite-containing medium. The isolated strains were identified based on full 16S rRNA gene sequences and partial *rpoB* gene sequences as *C*. *xerosis*, *C*. *variabile*, *C*. *lactis*, *C*. *callunae*, *C*. *confusum*, *C*. *glutamicum* and *C*. *crudilactis*. The identification based on 16S rRNA and *rpoB* sequences was not reliable for isolates of *C*. *xerosis*. Chemotaxonomic markers of the isolates, fatty acids, acyl type of peptidoglycan, presence and length of mycolic acids, quinone patterns, and polar lipids, were in accord with the known characteristics of these species. Biochemical profiles, analyzed with the API Coryne system, were able to differentiate all groups, but were unable to identify the strains due to an inappropriate database for raw-milk associated corynebacteria. Most of the tested isolates showed a single-substance resistance against oxacillin, but three single isolates were classified as multidrug resistant.

## Introduction

Species of the genus *Corynebacterium* were found ubiquitously in the environment, although often their natural habitat—especially the habitat of nonmedical *Corynebacterium* species—remains unknown [1]. In various studies, pathogenic corynebacteria were detected in raw milk samples and *Corynebacterium* spp. are known to cause subclinical mastitis in dairy cows [2]. *Corynebacterium bovis* is a common agent of bovine subclinical mastitis [3,4] and other species, e.g., *C*. *amycolatum*, *C*. *minutissimum*, *C*. *ulcerans* and *C*. *pseudotuberculosis*, were associated with clinical or subclinical bovine mastitis as well [5,6].

Non-pathogenic *Corynebacterium* species were also frequently isolated from raw milk or raw milk products [7,8]. Among them were also some species with beneficial functions in food processing. For example, the species *C*. *glutamicum* and *C*. *variabile* are well-known amino-acid producers [9] and the species *C*. *casei*, *C*. *mooreparkense*, *C*. *ammoniagenes* and *C*. *stationis*, have been detected on the surface of smear ripened cheese and are supposed to contribute to the flavor of the cheese [10,11].

The knowledge about corynebacterial diversity in raw milk is still fragmentary because of inappropriate routine test systems and high numbers of misidentifications [6,12]. For example, *C*. *xerosis* has been considered a serious and frequent human pathogen, until findings [12] indicated that most of the clinical isolates were misidentified strains of *C*. *amycolatum*. The identification to species level by analysis of their 16S rRNA gene sequences is sometimes not reliable because the 16S rRNA genes of some species show sequence differences below 2% [13,14]. For these species, the identification by additional genes has been proposed, such as *rpoB* gene sequencing [15]. Additionally, *Corynebacterium* species often grow weak on standard laboratory media. Most species show enhanced growth in sheep blood broth or brain-heart infusion, with 0.1–1.0% Tween 80 for the growth of lipophilic species [1,16]. Selective agars for *Corynebacterium* species are based on tellurite and have been described for *C*. *diphteriae* and *C*. *ulcerans* [17,18]. They are based on the ability of *Corynebacterium* species, among other Gram-positive species, to grow in the presence of tellurite, in contrast to most Gram-negative species [19].

The aim of our study was to isolate and characterize *Corynebacterium* spp. from bulk tank raw cow´s milk of different dairy farms and to illuminate potential pitfalls in the identification process. *Corynebacterium* species represent only a minor part of the raw milk microbiota [20]. Therefore, we evaluated the use of a selective medium for *Corynebacterium* species based on brain-heart infusion agar supplemented with tellurite and Tween 80 to detect also slow growing strains with minor abundance.

## Material and methods

### Evaluation of a selective medium for *Corynebacterium* spp.

The selective medium used in this study was based on brain-heart infusion (Oxoid Ltd., Hampshire, United Kingdom) solidified with 1.5% (w/v) agar (Oxoid Ltd., Hampshire, United Kingdom). Tween 80 (Merck KGaA, Darmstadt, Germany) was added in concentrations of 0.1% or 1.0% (w/v) [16]. Potassium tellurite trihydrate (Merck KGaA, Darmstadt, Germany) was dissolved in distilled water and added filter-sterilized to the autoclaved medium in concentrations of 0.15 g/L [21], 0.25 g/L [22] or 0.36 g/L [16]. Selectivity of this medium was tested with type strains and isolates of the genus *Corynebacterium* and with isolates of other non-target genera: *C*. *frankenforstense* ST18^T^, *C*. *lactis* RW2-5^T^, *C*. *glutamicum* DSM 20300^T^, *C*. *amycolatum* DSM 6922^T^, *C*. *camporealensis* NS1-11, *C*. *flavescens* TS21, *C*. *xerosis* M3_I15, *C*. *confusum* M3_I13, *C*. *casei* M3_I10, *Lactococcus lactis subsp*. *lactis* JZ RK-40, *Bacillus subtilis* M3_I11, *Staphylococcus chromogenes* M3_I12, *Escherichia coli* M3_I20, *Acinetobacter guillouiae* M3_I21 and *Pseudomonas gessardii* M3_I22. All isolates were identified based on their fatty acid profiles and 16S rRNA gene sequences [8] and except for *C*. *glutamicum* DSM 20300^T^ and *C*. *amycolatum* DSM 6922^T^, all strains were recovered from raw milk [8,23].

### Cultivation and isolation

Nine raw milk samples were collected from the bulk tank of seven dairy farms (farm B, K, M, N, P1, P2 and F) operated by private farmers, who gave their permission to conduct the study on their site. The dairy farms are located in the greater Bonn area in North Rhine-Westphalia, Germany. Ethical approval of the local authorities was not required because the raw milk samples were taken directly from the bulk tank, in accordance with the owners of the farms. Animals were not affected by the sampling procedure. The milk samples were cultivated on brain-heart infusion agar with 0.25 g/L potassium tellurite and 1.0% Tween 80 (BHT-agar) at 30°C for 48 h. Total bacterial counts were determined on Trypton soy agar (TSA; Merck KGaA, Darmstadt, Germany). Colonies from BHT-agar with bacterial cells of rod-shaped morphology were subcultivated on TSA as presumptive *Corynebacterium* spp.

### 16S rRNA and *rpoB* gene sequencing

Extraction of genomic DNA, amplification and sequencing of 16S rRNA genes was performed as described previously [23,24]. 16S rRNA genes were amplified with the universal bacterial primers 8F (5´-AGAGTTTGATCMTGGC-3´) and 1492R (5´-TACCTTGTTACGACTT-3´) and as sequencing primers we used 787R (5´-GGACTACCAGGGTATCTAAT-3´) and 518F (5´-CCAGCAGCCGCGGTAAT-3´) [24]. Amplification of partial *rpoB* genes was performed with the *Corynebacterium* specific primers C2700F (5´-CGWATGAACATYGGBCAGGT-3´) and C3130R (5´-TCCATYTCRCCRAARCGCTG-3´) [15]. These primers were also used as sequencing primers. The obtained sequences were manually edited with Chromas Lite 2.1.1. (Technelysium Pty Ltd, South Brisbane, AU) and assembled with BioEdit 7.2.5. [25] to obtain either almost complete 16S rRNA sequences of 1,400–1,500 base pairs (bp) or partial *rpoB* gene sequences (300–400 bp). The sequences were compared to the sequences of type strains by using the Basic local alignment search tool (BLAST) [26]. Phylogenetic trees of isolates, closest related type strains and other raw milk associated *Corynebacterium* species were obtained by maximum-likelihood algorithm with MEGA 6.06. [27] and the alignment of the sequences was performed by ClustalW [28]. Model parameters were estimated using the “find best DNA” option of MEGA and models were chosen according to lowest Bayesian information criterion (BIC) and Akaike information criterion (AIC) values [27]. As best fit substitution model for 16S rRNA gene sequences, the Tamura-3-parameter model was chosen with discrete gamma distribution and presence for invariant sites. The *rpoB* gene sequences were analyzed based on the Tamura-Nei model with discrete gamma distribution and presence for invariant sites. The nearest-neighbor-interchange search method was used for both trees.

### Chemotaxonomic and biochemical properties

#### Chemotaxonomic characteristics

Fatty acid patterns, presence and length of mycolic acids, polar lipid patterns, quinones and the acyl type of peptidoglycan were determined as described previously [23].

#### API Coryne test system

Biochemical tests were performed with the API Coryne test system (BioMèrieux, F) for identifying coryneform bacteria according to the manufacturer’s specifications. Microorganisms were cultured on TSA for 24 h at 30°C and the inoculated test strips were incubated for 24–48 h at 30°C. Reaction profiles were analyzed with the apiweb^TM^ software. The database version was V3.0.

#### Proteolytic and lipolytic activity

Proteolytic activity was determined on skim milk agar (TSA with 5.0% w/v skim milk powder) and lipolytic activity on tributyrin agar (TSA with 1.0% v/v tributyrin) [29]. Both tests were performed at 30°C for 48 h and at 10°C for 7 d. Strains were considered positive for proteolysis or lipolysis by formation of a transparent halo around the colonies.

### Susceptibility against antimicrobial agents

Susceptibility patterns against 16 antimicrobial agents of different classes were determined on Mueller Hinton agar (Oxoid Ltd., Hampshire, United Kingdom) by the agar disk diffusion method. All antimicrobial susceptibility disks were purchased from Oxoid (Hampshire, United Kingdom) or bestbion (Cologne, Germany). The antimicrobial agents used in this study were: penicillin G (6μg, Oxoid), oxacillin (1 μg, Oxoid), ampicillin (10 μg, Oxoid), tetracyclin (30 μg, bestbion), gentamicin (10 μg, bestbion), erythromycin (15 μg, bestbion), trimethoprim/sulfonamide (1.25/23.75 μg, bestbion), ceftiofur (30 μg, bestbion), cefazolin (30 μg, bestbion), cephalothin (30 μg, bestbion), pirlimycin (2 μg, bestbion), amoxicillin/clavulanic acid (20/10 μg, bestbion), kanamycin (30 μg, bestbion), streptomycin (10 μg, bestbion), tobramycin (30 μg, bestbion) and amikacin (30 μg, bestbion). Strains were considered resistant, intermediate or susceptible according to zone diameters of the CLSI document VET01-A4 [30].

## Results and discussion

### Evaluation of different selective media

All reference strains showed intense growth on brain-heart infusion agar without supplements. The addition of potassium tellurite inhibited the growth of the *Gammaproteobacteria* strains *Escherichia coli* M3_I20, *Acinetobacter guillouiae* M3_I21 and *Pseudomonas gessardii* M3_I22 at all concentrations. Colonies of the *Corynebacterium*, *Staphylococcus*, *Bacillus* and *Lactococcus* strains were visible after two days of incubation at 30°C. Earlier studies showed that Gram-negative species are especially sensitive against tellurite and its strongly oxidizing potential [31], whereas Gram-positive genera like *Staphylococcus*, *Enterococcus* and *Corynebacterium* are able to grow in the presence of tellurite [19,31]. The content of *Gammaproteobacteria* increases during the storage of raw milk in the bulk tank at approximately 4°C [20]. These organisms usually grow fast on standard media and may overgrow *Corynebacterium* strains, but are inhibited by tellurite.

Strains grown on tellurite formed black and small colonies. Growth of the *Corynebacterium*, *Staphylococcus*, *Bacillus* and *Lactococcus* reference strains was weaker at 0.36 g/L potassium tellurite than at 0.25 g/L or 0.15 g/L. Therefore, 0.25 g/L tellurite was used as additive for the selective cultivation of *Corynebacterium* spp. Neither 0.1% nor 1.0% Tween 80 had an enhancing or inhibiting effect on the growth of the non-lipophilic *Corynebacterium* reference strains. Because the supplementation of Tween 80 is essential for cultivation of lipophilic species, it was added at a concentration of 1.0% to the selective agar.

### Bacterial counts and isolation procedure

On BHT-agar, the mean number of bacterial counts in nine raw milk samples was 5.0 x 10^3^ cfu/mL (colony-forming unit) with a range from 1.0 x 10^2^ to 2.4 x 10^4^ cfu/mL. The mean number of bacterial counts on TSA was 8.1 x 10^4^ cfu/mL (range from 2.0 x 10^3^ to 2.7 x 10^5^ cfu/mL). The average ratio of bacterial counts on BHT-agar to total bacterial counts on TSA was 6.1% with a range from 1.2% to 165.0%. Colonies on BHT-agar were screened microscopically for a rod-shaped cell morphology and rods without endospores were subcultivated as presumptive *Corynebacterium* spp. These isolates (n = 68) were obtained from five raw milk samples taken from three dairy farms.

### Identification of raw milk isolates

#### Raw milk associated *Corynebacterium* species

The isolates were identified based on their partial or full 16S rRNA gene sequences and partial *rpoB* gene sequences and out of 68 isolates, 28 were identified as *Corynebacterium* spp. The other isolates were members of the genera *Brevibacterium*, *Microbacterium*, *Arthrobacter*, *Dietzia* or *Psychrobacillus*. The *Corynebacterium* isolates were assigned to six different species: *C*. *callunae*, *C*. *xerosis*, *C*. *variabile*, *C*. *confusum*, *C*. *glutamicum* and *C*. *lactis*. One isolate, JZ16^T^, could not be assigned to any of the known *Corynebacterium* species. For this strain, the new species *C*. *crudilactis* was proposed recently [32]. *Corynebacterium* isolates were recovered from raw milk of three different dairy farms; none were detected in the raw milk samples of the other four dairy farms. This confirms a report [33], in which *Corynebacterium* species were only detected in 25% of the analyzed raw milk samples. All of the identified *Corynebacterium* species in this study were recovered from raw milk before, except for *C*. *callunae*, and may be part of the natural raw milk microbiota [8,9,23]. *C*. *xerosis* was the only species isolated from raw milk of three different dairy farms and isolates of this species have frequently been recovered from raw cow´s milk [6,8]. As it is considered a commensal of the mammalian and bovine mucous membrane [34,35], it may contaminate raw milk as part of the cow’s natural udder microbiota. The isolated *Corynebacterium* species are considered as non-pathogenic and an impact on human health is unlikely. Except for *C*. *confusum*, which is a rare human pathogen and was rarely isolated from clinical material [36,37], but no data is available considering the pathogenicity of *C*. *confusum* in animals or the potential of a human infection via zoonotic transmission.

#### 16S rRNA and rpoB gene sequence analyses

**Fig 1:**
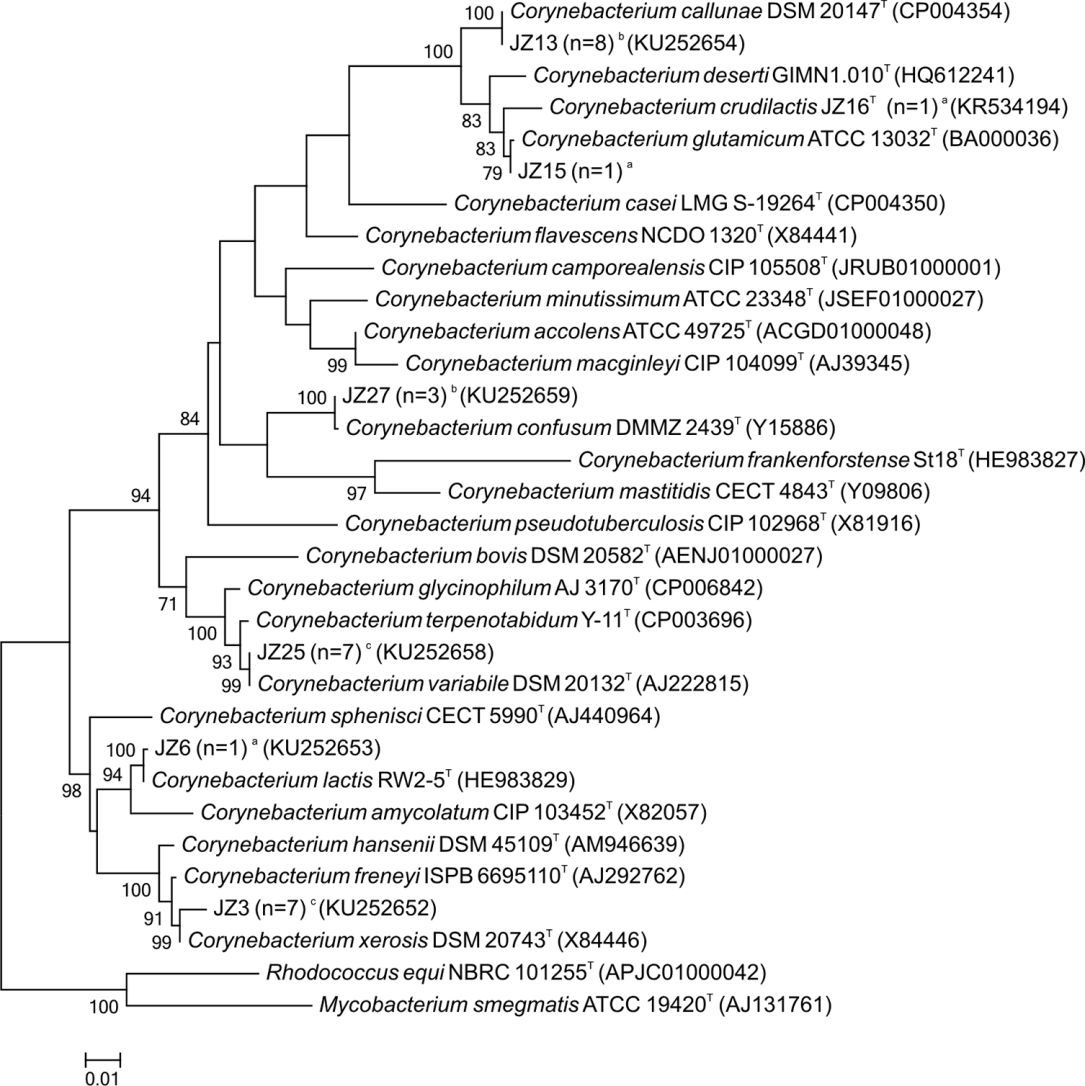
Phylogenetic relationships of representative isolates and related type strains from their 16S rRNA gene sequences. Bootstrap values greater than 70% based on 1,000 replicates are indicated at each node. Bar, 0.01 substitutions per nucleotide position. ^a^Isolated species detected in one sample of one dairy farm. ^b^Isolated species detected in more than one sample of one dairy farm. ^c^Isolated species detected in samples of more than one dairy farm.

**Fig 2:**
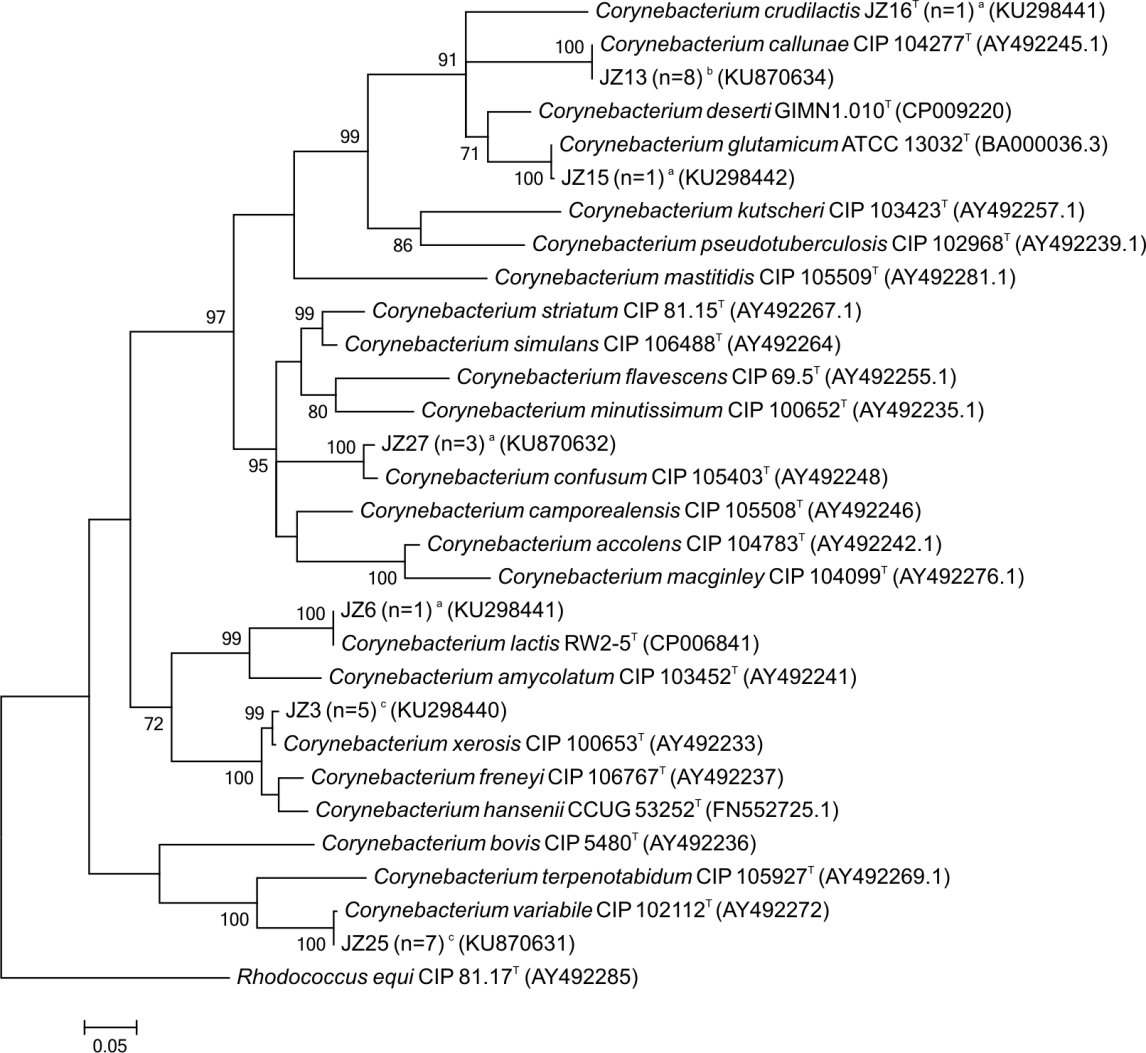
Phylogenetic relationships of representative isolates and related type strains from their *rpoB* gene sequences. Bootstrap values greater than 70% based on 1,000 replicates are indicated at each node. Bar, 0.05 substitutions per nucleotide position. ^a^Isolated species detected in one sample of one dairy farm. ^b^Isolated species detected in more than one sample of one dairy farm. ^c^Isolated species detected in samples of more than one dairy farm.

Results of 16S and *rpoB* gene sequencing are given in Table 1 and GenBank Accession numbers of the gene sequences in S1 Table. Phylogenetic relationships of the isolates and type strains based on maximum-likelihood analysis of their 16S rRNA or *rpoB* gene sequences are shown in Figs 1 and 2. The identification of *C*. *lactis*, *C*. *callunae*, and *C*. *confusum* was reliably based on a pairwise similarity of at least 99.7% to the 16S rRNA gene sequences of the type strains. Additionally, the pairwise similarity to the gene sequences of the next related type strain did not exceed the proposed threshold for species delineation of 97.8% [38]. The isolates identified as *C*. *variabile*, *C*. *xerosis*, *C*. *glutamicum* and *C*. *crudilactis* were not reliably differentiated from their next relatives by 16S rRNA gene sequencing and the deviation of their16S rRNA gene sequences ranged from 0.1–2.1%. Partial *rpoB* gene sequence analyses were needed to reliably identify the isolates of *C*. *variabile*, *C*. *glutamicum* and *C*. *crudilactis*. Here, the pairwise similarity to the next related type strain was below 90.2% and complied with the proposed cutoff for species delineation of 95.0% [39]. However, *C*. *xerosis* and the most closely related species *C*. *freneyi* were hardly distinguishable by *rpoB* gene sequencing as well. The deviation of their *rpoB* gene sequences ranged between 2.5 and 4.5% and the average pairwise similarity was 95.4% (94.9–95.8%). This supports findings [15] that the *rpoB* gene sequence similarity of *C*. *xerosis* and *C*. *freneyi* is the highest among all *Corynebacterium* species, which are closely related. For *C*. *xerosis* and *C*. *freneyi*, restriction length polymorphism analysis of the 16S-23S spacer region has been proposed to clearly differentiate the two species [40]. Additionally, multilocus sequence analyses of several housekeeping genes, e.g. *atpA*, *dnaA*, *fusA*, *odhA*, or whole genome sequencing can be applied to improve the resolution of phylogenetic relationships between these closely related *Corynebacterium* species [41].

**Table 1:** Pairwise similarity of 16S rRNA and *rpoB* gene sequences of the isolates and type strains.

Strains	Next-related type strain	% Similarity		
		16S rRNA		*rpoB*
		Partial	full	
JZ2	*Corynebacterium xerosis* DSM 20743^T^	100.0	99.5	100.0
JZ3	*Corynebacterium xerosis* DSM 20743^T^	100.0	99.4	98.6
JZ1	*Corynebacterium xerosis* DSM 20743^T^	100.0	-	99.5
JZ4	*Corynebacterium xerosis* DSM 20743^T^	100.0	-	99.8
JZ5	*Corynebacterium xerosis* DSM 20743^T^	100.0	-	-
JZ19	*Corynebacterium xerosis* DSM 20743^T^	100.0	-	-
N1	*Corynebacterium xerosis* DSM20743^T^	100.0	99.2	97.6
JZ6	*Corynebacterium lactis* RW2-5^T^	99.4	99.7	99.8
JZ20	*Corynebacterium varabile* DSM 20132^T^	100.0	100.0	99.5
JZ25	*Corynebacterium varabile* DSM 20132^T^	100.0	100.0	99.5
JZ10	*Corynebacterium varabile* DSM 20132^T^	100.0	-	99.5
JZ11	*Corynebacterium varabile* DSM 20132^T^	100.0	-	99.5
JZ21	*Corynebacterium varabile* DSM 20132^T^	100.0	-	99.5
JZ28	*Corynebacterium varabile* DSM 20132^T^	100.0	-	99.5
JZ29	*Corynebacterium varabile* DSM 20132^T^	100.0	-	99.5
JZ13	*Corynebacterium callunae* DSM 20147^T^	99.9	99.9	100.0
JZ14	*Corynebacterium callunae* DSM 20147^T^	99.9	99.9	100.0
JZ22	*Corynebacterium callunae* DSM 20147^T^	99.9	-	100.0
JZ23	*Corynebacterium callunae* DSM 20147^T^	99.9	-	100.0
JZ26	*Corynebacterium callunae* DSM 20147^T^	99.9	-	100.0
JZ32	*Corynebacterium callunae* DSM 20147^T^	99.7	-	100.0
JZ34	*Corynebacterium callunae* DSM 20147^T^	99.6	-	100.0
JZ36	*Corynebacterium callunae* DSM 20147^T^	99.6	-	100.0
JZ15	*Corynebacterium glutamicum* ATCC 13032^T^	99.8	99,9	99.5
JZ16	*Corynebacterium crudilactis* JZ16^T^	100.0	100.0	100.0
JZ27	*Corynebacterium confusum* DMMZ 2439^T^	100.0	99.9	98.1
FF1	*Corynebacterium confusum* DMMZ 2439^T^	99.9	99.9	98.1
FF3	*Corynebacterium confusum* DMMZ 2439^T^	99.9	99.9	98.4

### Chemotaxonomic and biochemical features of *Corynebacterium* isolates

#### Fatty acid pattern

All isolated *Corynebacterium* strains showed long-chain saturated and unsaturated fatty acids, as described for the genus *Corynebacterium* [1]. In contrast to other genera of the *Corynebacteriales*, the species of this genus contain no or low amounts of tuberculostearic acid [1]. Species-specific fatty acid patterns were detected for the seven identified *Corynebacterium* species (Table 2). The species showed quantitative and qualitative differences among one another, especially in the presence of minor compounds. For example, *C*. *lactis* JZ6 was clearly separated from the other strains because it contained minor amounts of the fatty acids C_17:1_ *cis* 9 (9.9%) and C_17:0_ (17.1%) and the strains of *C*. *variabile* contained little to moderate amounts (2.3–15.3%) of the diagnostic compound tuberculostearic acid (TBSA; C_18:0_ 10-methyl). Only few *Corynebacterium* species contain moderate amounts of TBSA, e.g. *C*. *variabile*, *C*. *ammoniagenes* and *C*. *bovis* [1]. Traces of TBSA were detected for *C*. *confusum* as well [36], but this could not be confirmed in this study. Fatty acid analyses may allow differentiation between *C*. *xerosis* and the closely related *C*. *freneyi*. According to Funke and Frodl [40], strains of *C*. *freneyi* contain little amounts of the unsaturated fatty acid C_17:1_ *cis* 8, which was not detected for *C*. *xerosis* in this study (Table 2). Additionally, *C*. *freneyi* strains contain lower levels of C_18:1_ *cis* 9 (21%) [40], compared to *C*. *xerosis*, where C_18:1_ *cis* 9 was the main fatty acid (66.8–85.3%; Table 2). The strains of *C*. *confusum* contained large amounts (18.6–46.5%) of an unidentified component (ECL 16,697) that even had, in one case, a higher percentage than the main fatty acid C_18:1_ *cis* 9. Mass spectra revealed these compounds as saturated and unsaturated aldehydes, which were presumptive pyrolysis products of the corynemycolic acids [42]. The fatty acid composition proved to be a useful feature for a differentiation of *Corynebacterium* species because each one of the seven different species showed a unique fatty acid profile. Additionally, the presence of diagnostic fatty acids, e.g. TBSA, which are only present in a few *Corynebacterium* species, enables a quick distinction between species.

**Table 2:** Fatty acid composition (with standard deviation in parentheses) of isolated strains.

Item	*C*. *xerosis*	*C*. *lactis*	*C*. *variabile*	*C*. *callunae*	*C*. *glutamicum*	*C*. *crudilactis*	*C*. *confusum*
**No. of isolates**	7	1	7	8	1	1	3
**FA (%)**							
**C**_**14: 0**_			0.2 (0.2)				
**ECL 14.926** ^**a**^				8.6 (4.7)	3.7		0.7 (0.4)
**C**_**15:0**_					0.2	0.6	
**C**_**16:1**_ ***cis* 7**			0.1 (0.1)				0.1 (0.0)
**C**_**16:0**_	3.0 (2.4)	8.4	39.9 (9.0)	36.9 (1.5)	39.4	26.8	16.3 (2.9)
**ECL 16.697** ^**a**^			3.9 (2.9)	21.0 (7.5)	8.5	3.4	29.8 (14.7)
**C**_**17:1**_ ***cis* 9**		9.9					
**ECL 16.938** ^**a**^			0.4 (0.5)	1.8 (0.6)	1.2	1.1	1.2 (0.7)
**C**_**17:0**_	0.1 (0.2)	17.1				1.6	
**ECL 17.373** ^**a**^				0.2 (0.5)			
**C**_**18:1**_ ***cis* 9**	78.0 (6.5)	46.4	48.7 (13.3)	56.4 (6.5)	46.8	65.7	48.7 (11.5)
**C**_**18:0**_	18.8 (5.1)	18.2	8.6 (4.7)		0.1	0.8	3.2 (1.6)
**C**_**18:0**_ **10-methyl**			6.8 (4.7)				

^a^Unknown compound with a specific equivalent chain length (ECL).

#### Mycolic acids, acyl type of peptidoglycan, quinones, polar lipid pattern

**Fig 3:**
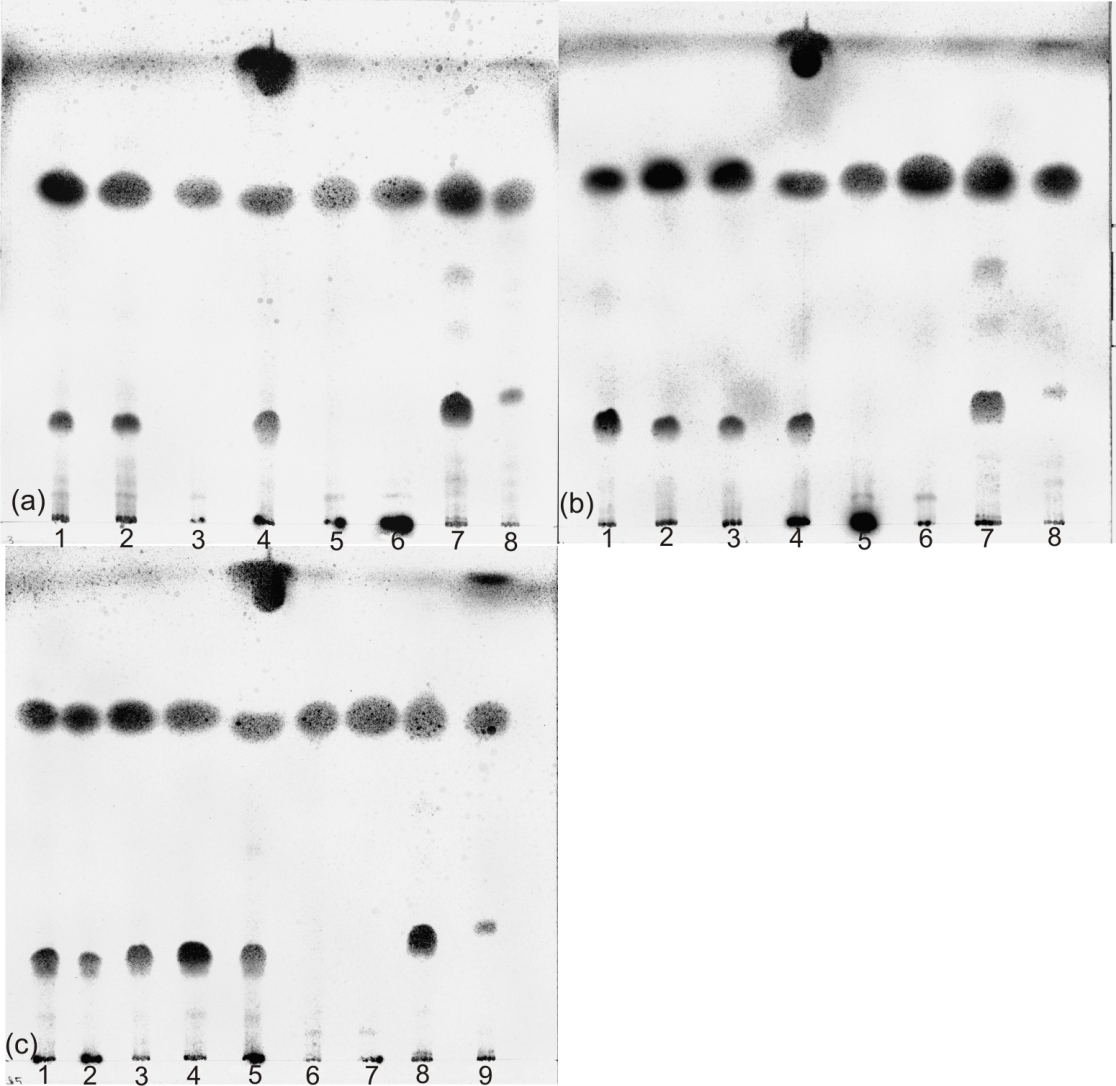
Mycolic acid analysis of isolates and reference strains of the suborder *Corynebacterineae*. Lanes of chromatogram (a): 1, *C*. *xerosis* JZ2; 2, *C*. *xerosis* JZ3; 3, *C*. *lactis* JZ6; 4, *C*. *glutamicum* DSM 20300^T^; 5, *C*. *amycolatum* DSM 6922^T^; 6, *C*. *lactis* RW 2-5^T^; 7, *Rhodococcus rhodochrous* DSM 43241^T^; 8, *Gordonia terrae* DSM 43249^T^. Lanes of chromatogram (b): 1, *C*. *confusum* JZ27; 2, *C*. *variabile* JZ20; 3, *C*. *variabile* JZ25; 4, *C*. *glutamicum* DSM 20300^T^; 5, *C*. *amycolatum* DSM 6922^T^; 6, *C*. *lactis* RW 2-5^T^; 7, *R*. *rhodochrous* DSM 43241^T^; 8, *G*. *terrae* DSM 43249^T^. Lanes of chromatogram (c): 1, *C*. *callunae* JZ13; 2, *C*. *callunae* JZ14; 3, *C*. *glutamicum* JZ15; 4, *C*. *crudilactis* JZ16^T^; 5, *C*. *glutamicum* DSM 20300^T^; 6, *C*. *amycolatum* DSM 6922^T^; 7, *C*. *lactis* RW 2-5^T^; 8, *R*. *rhodochrous* DSM 43241^T^; 9, *G*. *terrae* DSM 43249^T^.

**Fig 4:**
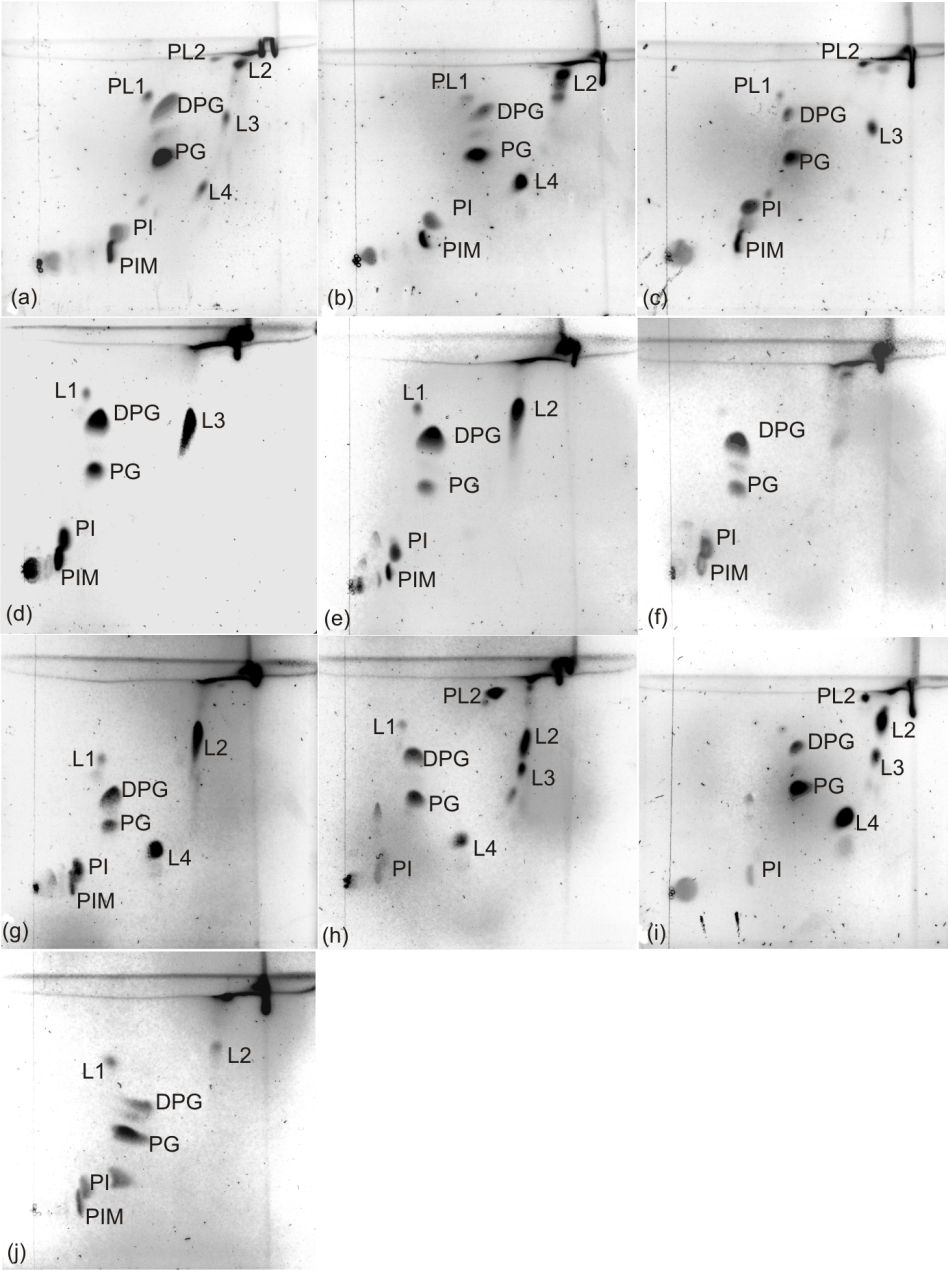
Polar lipid patterns of isolates. *C*. *xerosis* JZ2 (a), *C*. *xerosis* JZ3 (b), *C*. *lactis* JZ6 (c), *C*. *callunae* JZ13 (d), *C*. *callunae* JZ14 (e), *C*. *glutamicum* JZ15 (f), *C*. *crudilactis* JZ16^T^ (g), *C*. *variabile* JZ20 (h), *C*. *variabile* JZ25 (i), *C*. *confusum* JZ27 (j). DPG, diphosphatidylglycerol; PG, phosphatidylglycerol; PI, phosphatidylinositol; PIM, phosphatidylinositol mannoside; PL1 –PL2, phospholipids (molybdenum blue positive); L1 –L4, polar lipids (molybdenum blue negative).

A summary of the chemotaxonomic characteristics of representative isolates is given in Table 3. The isolated strains contained mycolic acids with a chromatographic mobility comparable to the mycolic acids of *C*. *glutamicum* DSM 20300^T^, as described for members of these species [1], except for strain *C*. *lactis* JZ6. *C*. *lactis* belongs, together with the species *C*. *amycolatum*, *C*. *caspium*, *C*. *ciconiae* and *C*. *kroppenstedtii*, to the small group of *corynebacteria* without mycolic acids [1,23]. Mycolic acids are long-chained, saturated and unsaturated, β-hydroxy fatty acids with a long α-alkyl branch, characteristically synthesized by members of the order *Corynebacteriales* [43]. They vary in structure, chain length and in the degree of unsaturation between the different genera of the order *Corynebacteriales* and allow a differentiation of the genus *Corynebacterium* that characteristically shows short mycolic acids with 22–38 carbons [1]. All strains showed the acetyl type of peptidoglycan. Dihydrogenated menaquinones with nine isoprene units [MK-9 (H_2_)] were detected as major menaquinones (> 60%) for all of the strains, except for the strains of *C*. *confusum*, which showed MK-8 (H_2_) as major menaquinone. The analysis of bacterial isoprenoid quinones and polar lipids is used for the characterization of corynebacteria and related genera [44,45,46]. The phospholipids diphosphatidylglycerol (DPG) and phosphatidylglycerol (PG) were detected in all of the strains. Phosphatidylinositol (PI) and phosphatidylinositol mannoside (PIM) were detected as well, but whereas PI was present in all strains, PIM was not detected in the strains of *C*. *variabile*. Aminolipids were not detected. This confirms data from earlier publications, where DPG, PG, PI and PIM were detected for *C*. *xerosis*, *C*. *glutamicum* and *C*. *lactis* [23,47,48]. Phospholipid patterns were not determined so far for *C*. *variabile*, *C*. *confusum* and *C*. *callunae*. PG and phosphatidylethanolamine (PE) are useful markers to differentiate corynebacteria from the related genera *Mycobacterium*, *Nocardia*, *Gordonia* and *Rhodococcus* [48]. PG was detected in substantial amounts only in *Corynebacterium* species and not in the genera *Mycobacterium*, *Nocardia* or *Gordonia* [48]. In contrast, PE is absent only in members of the genus *Corynebacterium*, but not in the other genera of the order *Corynebacteriales* [1, 48]. While DPG is a common compound in bacteria, PI was only detected in Actinomycetes and *Corynebacteriales*. Not all of the polar lipids could be identified in this study. Some strains contained molybdenum blue negative lipids with a high mobility in the second dimension. According to literature, this is characteristic for acidic glycolipids [49] and they have been detected for *C*. *xerosis* and *C*. *bovis* as well [48]. Thin-layer chromatograms of mycolic acids and polar lipids are given in Figs 3 and 4.

**Table 3:** Chemotaxonomic characteristics of representative isolates of each *Corynebacterium* species.

Species ^a^	Strains	FA pattern	Mycolic acids ^b^	Peptido-glycan-type	Menaquinones ^c^		Polar lipids ^d^
					+++	+	
***Corynebacterium xerosis***	JZ2, JZ3, N1	C_18:1_ *cis* 9, C_18:0_	coryne-mycolates	Acetyl	MK-9 (H_2_)	MK-8 (H_2_)	DPG, PG, PI, PIM
***Corynebacterium lactis***	JZ6	C_18:1_ *cis* 9, C_18:0_, 17:0, C_17:1_ *cis* 9	n.d.	Acetyl	MK-9 (H_2_)		DPG, PG, PI, PIM
***Corynebacterium variabile***	JZ20, JZ25	C_18:1_ *cis* 9, C_18:0_, C_18:0_ 10-methyl	coryne-mycolates	Acetyl	MK-9 (H_2_)	MK-8 (H_2_)	DPG, PG, PI
***Corynebacterium callunae***	JZ13, JZ14	C_18:1_ *cis* 9, C_16:0_	coryne-mycolates	Acetyl	MK-9 (H_2_)	MK-8 (H_2_)	DPG, PG, PI, PIM
***Corynebacterium glutamicum***	JZ15	C_18:1_ *cis* 9, C_16:0_, C_15:0_	coryne-mycolates	Acetyl	MK-9 (H_2_)	MK-8 (H_2_)	DPG, PG, PI, PIM
***Corynebacterium crudilactis***	JZ16^T^	C_18:1_ *cis* 9, C_15:0_, C_16:0_, C_17:0_	coryne-mycolates	Acetyl	MK-9 (H_2_)	MK-8 (H_2_)	DPG, PG, PI, PIM
***Corynebacterium confusum***	JZ27, FF1, FF3	C_18:1_ *cis* 9, C_17:0_	coryne-mycolates	Acetyl	MK-8 (H_2_)	MK-9 (H_2_)	DPG, PG, PI, PIM

^a^Identification according to 16S rRNA and *rpoB* gene sequences.

^b^Mycolic acids: n.d. = no Mycolic acids detected; corynemycolates = Mycolic acids with mobility identical with those of *Corynebacterium glutamicum* DSM 20300^T^ using thin-layer chromatography.

^c^Menaquinones: +++ = main component (>60%); + = minor component (<40%).

^d^Polar lipids: DPG = diphosphatidylglycerol. PG = phosphatidylglycerol; PI = phosphatidylinositol; PIM = phosphatidylinositol mannoside.

#### API Coryne

Identification of *Corynebacterium* species by the API Coryne test system is a fast and easy method and earlier studies showed that the numbers of misidentifications of clinical isolates are relatively low [50,51,52]. Results of the API Coryne test system for our isolates are shown in Table 4. The numerical code was obtained after an incubation period of 24 h. Test strips of the strains of *C*. *xerosis* and *C*. *confusum* were incubated 48 h because of their weak growth after 24 h. All of the tested strains were positive for pyrazinamidase and negative for gelatinase, pyrolidonyl arylamidase, N-acetyl-β-glucosaminidase and fermentation of glycogen and xylose. The numerical code was identical for the strains within the species *C*. *variabile*, *C*. *callunae* and *C*. *confusum* (Table 4), the three strains of *C*. *xerosis* showed different results in the API Coryne test. Two of the three tested strains of *C*. *xerosis* (JZ2 and JZ3) were negative for α-glucosidase activity and two strains (JZ3 and N1) were negative for alkaline phosphatase. This may also differentiate the isolated *C*. *xerosis* strains from the closely related *C*. *freneyi*. Strains of *C*. *freneyi* are described consistently positive for α-glucosidase activity and alkaline phosphatase [40]. None of the isolates were correctly identified by this test system, which is explained by the lack of these raw milk associated species in the present database version. Therefore, correct identification of milk-associated *Corynebacterium* species is critical with this system.

**Table 4:** Numerical code of representative isolates generated with the API Coryne test system.

Species ^a^	Strain	Numerical code	Apiweb-Identification ^d^	
			Sign. Taxa	% ID (T)
***Corynebacterium xerosis*** ^**b**^	JZ2	3500365	Incorrect profile ^c^	
	JZ3	2000325	*Corynebacterium* group G	48.5 (0.72)
	N1	2410344	*Actinomyces neuii* ssp. *anitratus*	95.0 (0.46)
***Corynebacterium lactis***	JZ6	2100304	*C*. *jeikeium*	93.6 (1.0)
***Corynebacterium callunae***	JZ13	2000325	*Corynebacterium* group G	48.5 (0.72)
	JZ14	2000325	*Corynebacterium* group G	48.5 (0.72)
***Corynebacterium glutamicum***	JZ15	3201325	*C*. *glucuronolyticum*	98.3 (0.92)
***Corynebacterium crudilactis***	JZ16^T^	3241304	*C*. *glucuronolyticum*	94.6 (0.72)
***Corynebacterium variabile***	JZ20	2011004	*C*. *urealyticum*	82.4 (0.67)
	JZ25	2011004	*C*. *urealyticum*	82.4 (0.67)
***Corynebacterium confusum*** ^**b**^	JZ27	3100304	*C*. *propinquum*	83.4 (1.0)
	FF1	3100304	*C*. *propinquum*	83.4 (1.0)
	FF3	3100304	*C*. *propinquum*	83.4 (1.0)

^a^Identification according to 16S rRNA and *rpoB* gene sequences.

^b^Incubation of test strips for 48 h.

^c^Incorrect profile indicated by the apiweb^TM^ Software.

^d^ Strains were subcultured on TSA for 24 h at 30°C and results were obtained after 24 h incubation of the test strips at 30°C.

#### Proteolytic and lipolytic activity

In order to determine the potential as food spoiling organisms, proteolytic and lipolytic activity of the *Corynebacterium* isolates were tested at 30°C and 10°C. None of the *Corynebacterium* strains showed proteolytic activity. Lipolytic activity was detected for the strains of *C*. *lactis*, *C*. *callunae*, *C*. *xerosis* and *C*. *variabile* at 30°C and the strains of *C*. *variabile* were able to perform lipolysis at 10°C as well. Although growth below 20°C is rarely detected within the genus *Corynebacterium* [1], all raw milk isolates, except strains of the species *C*. *confusum*, were able to grow at 10°C on TSA within 48 h.

### Antimicrobial susceptibility of *Corynebacterium* isolates

Nine selected strains were tested for susceptibility against 16 different antimicrobial agents: *C*. *lactis* JZ6, *C*. *callunae* JZ13, *C*. *glutamicum* JZ15, *C*. *crudilactis* JZ16^T^, *C*. *variabile* JZ20, *C*. *confusum* JZ27 and *C*. *xerosis* JZ2, JZ3 and N1. All of the strains were resistant against oxacillin. Additionally, *C*. *callunae* JZ13 was resistant against pirlimycin and *C*. *glutamicum* JZ15 against streptomycin. The three isolates *C*. *xerosis* N1, *C*. *confusum* JZ27 and *C*. *crudilactis* JZ16^T^ [32] were resistant against antimicrobial agents of three different classes, which qualified them as multidrug resistant (MDR) according to the definition of the CLSI [30]. *C*. *xerosis* N1 and *C*. *confusum* JZ27 were resistant against oxacillin, erythromycin and pirlimycin and *C*. *crudilactis* JZ16^T^ was resistant against oxacillin, ampicillin, thrimethoprim/sulfonamide, kanamycin and streptomycin [32]. Multidrug resistance has been described for clinically relevant *Corynebacterium* species, e.g. *C*. *resistens*, *C*. *striatum* or *C*. *amycolatum*, but rarely for non-medical corynebacteria [53,54,55]. Susceptibilities of raw milk associated *Corynebacterium* isolates against antimicrobial agents has not been described so far, expect for *C*. *bovis* and *C*. *amycolatum* isolates from bovine mammary glands [56]. Here, the *Corynebacterium* isolates were generally susceptible against the 15 tested antimicrobial agents. This supports findings of this study that antimicrobial resistance of raw milk associated corynebacteria and the potential to distribute antimicrobial resistance is generally low, except for single strains with high levels of antimicrobial resistance.

## Conclusion

Results of this study confirm that *Corynebacterium* species are a minor but regular part of the raw milk microbiome. Additionally, strains of other genera of the phylum *Actinobacteria* were isolated from raw milk on the selective medium in this study, e.g. *Arthrobacter* and *Dietzia*, which are rarely described as raw milk associated bacteria. Abundance and impact on raw milk of these organisms may need further investigation. A delineation of *Corynebacterium* spp. from other closely related genera of the order *Corynebacteriales* is possible by chemotaxonomic markers. Some of these markers, especially fatty acid profiles or the presence or absence of mycolic acids could also be used to differentiate between several milk-associated species within this genus. Sequencing of the 16S rRNA gene is appropriate for the identification of most *Corynebacterium* strains. For some species, a reliable identification needs additional sequence information from a less conserved gene like the *rpoB* gene, but for some closely related species, like *C*. *xerosis* and *C*. *freneyi*, additional tests (fatty acid pattern, α-glucosidase activity and alkaline phosphatase) are highly recommended. The high physiological diversity within the genus *Corynebacterium*, covering amino-acid producers, colonizers of smear-ripened cheese but also animal and human pathogens, gives reasons for further in-depth analyses of raw milk associated *Corynebacterium* species.

## Supporting information

S1 Table Accession numbers of the sequences used in this study. (PDF)

## References

[1] Bernard KA, Funke G. Genus I. *Corynebacterium* Lehmann and Neumann 1896, 350^AL^ emend. Bernard, Wiebe, Burdz, Reimer, Ng. Singh, Schindle and Pacheo 2010, 877 In: Goodfellow M, Kämpfer P, Busse HJ, Trujillo ME, Suzuki K, Ludwig W, Whitman WB, editors. Bergey’s Manual of Systematic Bacteriology. New York: Springer; 2012 pp. 245–289.

[2] Gonçalves JL, Tomazi T, Barreiro JR, Beuron DC, Arcari MA, Lee SHI, et al Effects of bovine subclinical mastitis caused by *Corynebacterium* spp. on somatic cell count, milk yield and composition by comparing contralateral quarters. Vet J. 2016; 209: 87–92. doi: 10.1016/j.tvjl.2015.08.009 26831159 10.1016/j.tvjl.2015.08.009

[3] Haltia L, Honkanen-Buzalski T, Spiridonova I, Olkonen A, Myllys V. A study of bovine mastitis, milking procedures and management practices on 25 Estonian dairy herds. Acta Vet Scand. 2006; 48: 22 doi: 10.1186/1751-0147-48-22 17118211 10.1186/1751-0147-48-22 PMC1664578

[4] Schukken YH, González RN, Tikofsky LL, Schulte HF, Santisteban CG, Welcome FL, et al CNS mastitis: Nothing to worry about? Vet Microbiol. 2009; 134: 9–14. doi: 10.1016/j.vetmic.2008.09.014 18842362 10.1016/j.vetmic.2008.09.014

[5] Hommez J, Devriese LA, Vaneechoutte M, Riegel P, Butaye P, Haesebrouck F. Identification of nonlipophilic Corynebacteria isolated from dairy cows with Mastitis. J Clin Microbiol. 1999; 37: 954–957. 10074508 10.1128/jcm.37.4.954-957.1999 PMC88631

[6] Watts JL, Lowery DE, Teel JF, Rossbach S. Identification of *Corynebacterium bovis* and other Coryneforms isolated from bovine mammary glands. J Dairy Sci. 2000; 83: 2373–2379. doi: 10.3168/jds.S0022-0302(00)75126-5 11049082 10.3168/jds.S0022-0302(00)75126-5

[7] Dolci P, Barmaz A, Zenato S, Pramotton R, Alessandrina V, Cocolin L, et al Maturing dynamics of surface microflora in Fontina PDO cheese studied by culture-dependent and–independent methods. J Appl Microbiol. 2009; 106: 278–287. doi: 10.1111/j.1365-2672.2008.04001.x 19054234 10.1111/j.1365-2672.2008.04001.x

[8] Weber M, Geißert J, Kruse M, Lipski A. Comparative analysis of bacterial community composition in bulk tank raw milk by culture-dependent and culture-independent methods using the viability dye propidium monoazide. J Dairy Sci. 2014; 97: 6761–6776. doi: 10.3168/jds.2014-8340 25242425 10.3168/jds.2014-8340

[9] Fricker M, Skanseng B, Rudi K, Stessl B, Ehling-Schulz M. Shift from farm to dairy tank milk microbiota revealed by a polyphasic approach is independent from geographical origin. Int J Food Microbiol. 2011; 145: 24–30. 10.1016/j.ijfoodmicro.2010.08.025 20855121

[10] Bockelmann W, Hoppe-Seyler T. The surface flora of bacterial smear-ripened cheeses from cow´s and goat´s milk. Int Dairy J. 2001; 11: 307–314.

[11] Brennan NM, Brown R, Goodfellow M, Ward AC, Beresford BT, Simpson PJ, et al *Corynebacterium mooreparkense* sp. nov. and *Corynebacterium casei* sp. nov., isolated from the surface of a smear-ripened cheese. Int J Syst Evol Microbiol. 2001; 51: 843–852. doi: 10.1099/00207713-51-3-843 11411705 10.1099/00207713-51-3-843

[12] Funke G, Lawson PA, Bernard KA, Collins MD. Most *Corynebacterium xerosis* strains identified in the routine clinical laboratory correspond to *Corynebacterium amycolatum*. J Clin Microbiol. 1996; 34: 1124–1128. 8727888 10.1128/jcm.34.5.1124-1128.1996 PMC228967

[13] Renaud FN, Aubel D, Riegel P, Meugnier H, Bollet C. *Corynebacterium freneyi* sp. nov., α-glucosidase-positive strains related to *Corynebacterium xerosis*. Int J Syst Evol Microbiol. 2001; 51: 1723–1728. doi: 10.1099/00207713-51-5-1723 11594602 10.1099/00207713-51-5-1723

[14] Renaud FN, Le Coustumier A, Wilhem N, Aubel D, Riegel P, Bollet C, et al *Corynebacterium hansenii* sp. nov., an α-glucosidase-negative bacterium related to *Corynebacterium xerosis*. Int J Syst Evol Microbiol. 2007; 57: 1113–1116. doi: 10.1099/ijs.0.64665-0 17473268 10.1099/ijs.0.64665-0

[15] Khamis A, Raoult D, La Scola B. *RpoB* gene sequencing for identification of *Corynebacterium* species. J Clin Microbiol. 2004; 42: 3925–3931. doi: 10.1128/JCM.42.9.3925-3931.2004 15364970 10.1128/JCM.42.9.3925-3931.2004 PMC516356

[16] Riegel P, de Briel D, Prévost G, Jehl F, Monteil H. Genomic diversity among *Corynebacterium jeikeium* strains and comparison with biochemical characteristics and antimicrobial susceptibilities. J Clin Microbiol. 1994; 32: 1860–1865. 7989533 10.1128/jcm.32.8.1860-1865.1994 PMC263892

[17] Segal E, Eylan E. Cystine–Serum–Tellurite: A differential medium for *Corynebacterium diphteriae*. Med Microbiol Immunol. 1973; 158: 165–169. 4632985 10.1007/BF02120551

[18] Tinsdale GFW. A new medium for the isolation and identification of *C*. *diphteriae* based on the production of hydrogen sulphide. J Pathol Bacteriol. 1947; 59: 461–466.

[19] Von Graevenitz A, Bernard K. Genus Corynebacterium–Medical In: Dworkin M, Falkow S, Rosenberg E, Schleifer K-H, Stackebrandt E, editors. *The Prokaryotes*. New York: Springer; 2006 pp. 819–842.

[20] Raats D, Offek M, Minz D, Halpern M. Molecular analysis of bacterial communities in raw cow milk and the impact of refrigeration on its structure and dynamics. Food Microbiol. 2011; 28: 465–471. doi: 10.1016/j.fm.2010.10.009 21356452 10.1016/j.fm.2010.10.009

[21] Baird-Parker AC. An improved diagnostic and selective medium for isolating coagulase-positive Staphylococci. J Appl Bacteriol. 1962; 25: 12–19.

[22] Zebovitz E, Evans JB, Niven CF. Tellurite-Glycine Agar: a selective plating medium for the quantitative detection of coagulase-positive Staphylococci. J Bacteriol. 1955; 70: 686–690. 13271314 10.1128/jb.70.6.686-690.1955 PMC386272

[23] Wiertz R, Schulz SC, Müller U, Kämpfer P, Lipski A. *Corynebacterium frankenforstense* sp. nov. and *Corynebacterium lactis* sp. nov., isolated from raw cow milk. Int J Syst Evol Microbiol. 2013; 63: 4495–4501. doi: 10.1099/ijs.0.050757-0 23907219 10.1099/ijs.0.050757-0

[24] Buchholz-Cleven BEE, Rattunde B, Straub KL. Screening for genetic diversity of isolates of anerobic Fe(II)–oxidizing bacteria using DGGE and whole-cell hybridization. Syst Appl Microbiol. 1997; 20: 301–309.

[25] Hall TA. Bioedit: a user-friendly biological sequence alignment editor and analysis program for Windows 95/98/NT. Nucleic Acids Symp Ser. 1999; 41: 95–98.

[26] Altschul SF, Gish W, Miller W, Myer EW, Lipman DJ. Basic local alignment search tool. J Mol Biol. 1990; 215: 403–410. doi: 10.1016/S0022-2836(05)80360-2 2231712 10.1016/S0022-2836(05)80360-2

[27] Tamura K, Peterson D, Peterson N, Stecher G, Nei M, Kumar S. MEGA5: molecular evolutionary genetics analysis using maximum likelihood, evolutionary distance, and maximum parsimony methods. Mol Biol Evol. 2011; 28: 2731–2739. doi: 10.1093/molbev/msr121 21546353 10.1093/molbev/msr121 PMC3203626

[28] Thompson JD, Higgins DG, Gibson TJ. CLUSTAL W: improving the sensitivity of progressive multiple sequence alignment through sequence weighting, position-specific gap penalties and weight matrix choice. Nucleic Acids Res. 1994; 22: 4673–4680. 7984417 10.1093/nar/22.22.4673 PMC308517

[29] Harrigan WF. Laboratory Methods in Food Microbiology. 3rd edn. San Diego: Academic Press; 1998.

[30] CLSI. Performance standards for antimicrobial disk and dilution susceptibility test for bacteria isolated from animals 4th ed. Wayne: Clinical and Laboratory Standards Institute; 2013.

[31] Walter EG, Taylor DE. Plasmid-mediated resistance to tellurite: expressed and cryptic. Plasmid. 1992; 27: 52–64. 1741460 10.1016/0147-619x(92)90006-v

[32] Zimmermann J, Rückert C, Kalinowski J, Lipski A. *Corynebacterium crudilactis* sp. nov., a new *Corynebacterium* species isolated from raw cow´s milk. Int J Syst Evol Microbiol. 2016; 66: 1–6. 27666312 10.1099/ijsem.0.001509

[33] Vacheyrou M, Normand A-C, Guyot P, Cassagne C, Piarroux R, Bouton Y. Cultivable microbial communities in raw cow milk and potential transfers from stables of sixteen French farms. Int J Food Microbiol. 2011; 146: 253–262. doi: 10.1016/j.ijfoodmicro.2011.02.033 21429612 10.1016/j.ijfoodmicro.2011.02.033

[34] Coyle MB, Lipsky BA. Coryneform bacteria in infectious diseases: clinical and laboratory aspects. Clin Microbiol Rev. 1990; 3: 227–246. 2116939 10.1128/cmr.3.3.227 PMC358157

[35] Gonçalves JL, Tomazi T, Barreiro JR, Braga PA, Ferreira CR, Araújo Junior JP, et al Identification of Corynebacterium spp. isolated from bovine intramammary infections by matrix-assisted laser desorption ionization-time of flight mass spectrometry. Vet Microbiol. 2014; 173: 147–151. doi: 10.1016/j.vetmic.2014.06.028 25086477 10.1016/j.vetmic.2014.06.028

[36] Funke G, Osorio CR, Frei R, Riegel P, Collins MD. *Corynebacterium confusum* sp. nov., isolated from human clinical specimens. Int J Syst Bacteriol. 1998; 48: 1291–1296. doi: 10.1099/00207713-48-4-1291 9828429 10.1099/00207713-48-4-1291

[37] Bernard KA, Munro C, Wiebe D, Ongsansoy E. Characteristics of rare or recently described *Corynebacterium* species recovered from human clinical material in Canada. J. Clin. Microbiol. 2002; 40: 4375–4381. doi: 10.1128/JCM.40.11.4375-4381.2002 12409436 10.1128/JCM.40.11.4375-4381.2002 PMC139690

[38] Kim M, Oh H-S, Park S-C, Chun J. Towards a taxonomic coherence between average nucleotide identity and 16S rRNA gene sequence similarity demarcation of prokaryotes. Int J Syst Evol Microbiol. 2014; 64: 346–351. doi: 10.1099/ijs.0.059774-0 24505072 10.1099/ijs.0.059774-0

[39] Khamis A, Raoult D, La Scola B. Comparison between *rpoB* and 16S rRNA gene sequencing for molecular identification of 168 clinical isolates of *Corynebacterium*. J Clin Microbiol. 2005; 43: 1934–1936. doi: 10.1128/JCM.43.4.1934-1936.2005 15815024 10.1128/JCM.43.4.1934-1936.2005 PMC1081344

[40] Funke G, Frodl R. Comprehensive study of *Corynebacterium freneyi* strains and extended and emended description of *Corynebacterium freneyi* Renaud, Aubel, Riegel, Meugnier, and Bollet 2001. J Clin Microbiol. 2008; 46: 638–643. doi: 10.1128/JCM.01491-07 18077628 10.1128/JCM.01491-07 PMC2238093

[41] König C, Meinel DM, Margos D, Konrad R, Sing A. Multilocus sequence typing of *Corynebacterium ulcerans* provides evidence for zoonotic transmission and for increased prevalence of certain sequence types among toxigenic strains. J. Clin. Microbiol. 2002; 52: 4318–4324. 10.1128/JCM.02291-14 PMC4313305 25320226

[42] Lechevalier MP, Lechevalier H, Horan AC. Chemical characteristics and classification of nocardiae. Can J Microbiol. 1973; 19: 965–972. 4752341 10.1139/m73-154

[43] Puech V, Chami M, Lemassu A, Lanéelle M-A, Schiffler B, Gounon P, et al Structure of the cell envelope of Corynebacteria: importance of the non-covalently bound lipids in the formation of the cell wall permeability barrier and fracture plane. Microbiology. 2001; 147: 1365–1382. doi: 10.1099/00221287-147-5-1365 11320139 10.1099/00221287-147-5-1365

[44] Bendinger B, Kroppenstedt RM, Klatte S, Altendorf K. Chemotaxonomic differentiation of coryneform bacteria isolated from biofilters. Int J Syst Bacteriol. 1992; 42: 474–486. doi: 10.1099/00207713-42-3-474 1503976 10.1099/00207713-42-3-474

[45] Brennan PJ, Lehane DP. The Phospholipids of Corynebacteria. Lipids. 1971; 6: 401–409. 4330104 10.1007/BF02531377

[46] Yamada Y, Inouye G, Tahara Y, Kondô K. The menaquinone system in the classification of cornyeform and nocardioform bacteria and related organisms. J Gen Appl Microbiol. 1976; 22: 203–214.

[47] Hoischen C, Krämer R. Membrane alteration is necessary but not sufficient for effective glutamate secretion in *Corynebacterium glutamicum*. J Bacteriol. 1990; 172: 3409–3416. 1971623 10.1128/jb.172.6.3409-3416.1990 PMC209152

[48] Minnikin DE, Patel PV, Alshamaony L, Goodfellow M. Polar lipid composition in the classification of *Nocardia* and related bacteria. Int J Syst Bacteriol. 1977; 27: 104–117.

[49] Wilkinson SG. Cell walls of *Pseudomonas* species sensitive to ethylendiaminetetraacetic acid. J Bacteriol. 1970; 104: 1035–1044. 16559075 10.1128/jb.104.3.1035-1044.1970 PMC248259

[50] Konrad R, Berger A, Huber I, Boschert V, H'rmansdorfer S, Busch U, et al Matrix-assisted laser desorption/ionization time-of-flight (MALDI-TOF) mass spectrometry as a tool for rapid diagnosis of potentially toxigenic *Corynebacterium* species in the laboratory management of diphtheria-associated bacteria. Euro Surveill. 2010; 15: 43. 10.2807/ese.15.43.19699-en 21087580

[51] Renaud FN, Dutaur M, Daoud S, Aubel D, Riegel P, Monget D, et al Differentiation of *Corynebacterium amycolatum*, *C*. *minutissimum*, and *C*. *striatum* by Carbon Substrate Assimilation Tests. J Clin Microbiol. 1998; 36: 3698–3702. 9817901 10.1128/jcm.36.12.3698-3702.1998 PMC105268

[52] Soto A, Zapardiel J, Soriano F. Evaluation of API Coryne system for identifying coryneform bacteria. J Clin Pathol. 1994; 47: 756–759. 7962633 10.1136/jcp.47.8.756 PMC502153

[53] Bernard KA. The genus *Corynebacterium* and other medically relevant Coryneform-like bacteria. J Clin Microbiol. 2012; 50: 3152–3158. doi: 10.1128/JCM.00796-12 22837327 10.1128/JCM.00796-12 PMC3457441

[54] De Miguel-Martinez I, Fernandez-Fuertes F, Ramos-Macias A, Bosch-Benitezand JM, Martin-Sanchez AM. Sepsis due to multiply resistant *Corynebacterium amycolatum*. Eur J Clin Microbiol. 1996; 15: 617–618. 10.1007/BF01709376 8874085

[55] Otsuka Y, Kawamura Y, Koyama T, Iihara H, Ohkusu K, Ezaki T. *Corynebacterium resistens* sp. nov., a new multidrug-resistant coryneform bacterium isolated from human infections, J Clin Microbiol, 2005; 43: 3713–3717. doi: 10.1128/JCM.43.8.3713-3717.2005 16081900 10.1128/JCM.43.8.3713-3717.2005 PMC1233995

[56] Watts JL, Rossbach S. Susceptibilities of *Corynebacterium bovis* and *Corynebacterium amycolatum* isolates from bovine mammary glands to 15 antimicrobial agents. Antimicrob Agents Chemother. 2000; 44: 3476–3477. 11083663 10.1128/aac.44.12.3476-3477.2000 PMC90228

