# Supplementary material for: Isolation and characterization of Corynebacterium spp. from bulk tank raw cow's milk of different dairy farms in Germany
Source: PLoS One. 2018 Apr 4;13(4):e0194365. doi: 10.1371/journal.pone.0194365 (PMC5884509; doi:10.1371/journal.pone.0194365)
Supplement: S1 Table — (PDF) [file pone.0194365.s001.pdf]

S1 Table. Accession numbers of the sequences used in this study.

Strains	GenBank Accession numbers	
	**16S rRNA**	***rpoB*** [c]
JZ1	KX359581 [b]	KY013617
JZ2	KU252651 [a]	KU298439
JZ3	KU252652 [a]	KU298440
JZ4	KX359591 [b]	KY013618
JZ5	KX359592 [b]	-
JZ6	KU252653 [a]	KU870633
JZ10	KX359583 [b]	KX965686
JZ11	KX359582 [b]	KX965687
JZ13	KU252654 [a]	KU870634
JZ14	KU252655 [a]	KU870629
JZ15	KU252656 [a]	KU298442
JZ16^T	KR534194 [a]	KU298441
JZ19	KX359590 [b]	-
JZ20	KU252657 [a]	KU870630
JZ21	KX359584 [b]	KX965688
JZ22	KX359589 [b]	KX965689
JZ23	KX359586 [b]	KX965690
JZ25	KU252658 [a]	KU870631
JZ26	KX359585 [b]	KX965691
JZ27	KU252659 [a]	KU870632
JZ28	KX359588 [b]	KX965692
JZ29	KX359587 [b]	KX965693
JZ32	KX359593 [b]	KX965694
JZ34	KX359594 [b]	KX965695
JZ36	KX359595 [b]	KX965696
N1	KU252662 [a]	KU298443
FF1	KU252600 [a]	KX965697
FF3	KU252661 [a]	KX965698

[a] Complete 16S rRNA gene sequences (1,400 – 1,500 bp).

[b] Partial 16S rRNA gene sequences (600 – 800 bp).

[c] Partial *rpoB* gene sequences (300 – 400 bp).
